# Knowledge, perception and cervical cancer screening practices among nurses and midwives: a case study of Our Lady of Grace Hospital in the Asikuma Odoben Brakwa district, Central region-Ghana

**DOI:** 10.4314/ahs.v23i4.53

**Published:** 2023-12

**Authors:** Esther Selasi Avinu, Juliana Kutah, Prince Osei Akumiah, Kwabena Opoku-Addai

**Affiliations:** 1 Department of Nursing and Midwifery, Presbyterian University, Ghana. Asante-Akyem Agogo, Ghana, West Africa; 2 Department of Physician Assistantship, Presbyterian University, Ghana. Asante-Akyem Agogo, Ghana, West Africa

**Keywords:** Cervical cancer screening, l female healthcare workers, Ghana

## Abstract

**Background:**

Cervical cancer prevention can be achieved through comprehensive programs involving education, awareness creation, vaccination, screening, and early treatment. Health workers have a vital role to play in achieving this. Hence, they must be adequately equipped with the requisite knowledge of the condition since they provide information to their clients.

**Objective:**

This study determined the knowledge, perception, and screening practices on cervical cancer among female nurses and midwives in the Central Region of Ghana.

**Method:**

A quantitative descriptive cross-sectional survey amongst nurses and midwives was used for the study. A convenience sampling technique was applied to yield a representative sample of 130 female nurses and midwives working in Our Lady of Grace Hospital in the Central Region, Ghana. Data was collected from the participants using a self-administered questionnaire. The data was analysed using SPSS version 20, and standard descriptive statistics such as proportions were used to summarize the survey data.

**Results:**

Almost all the nurses (99%) had heard of cervical cancer. Majority (97.1%) of the respondents believed cervical cancer is preventable however, 67.6% also believed that it is incurable. Almost half (42.2%) of the respondents did not perceive themselves as at risk of cervical cancer. Thus, only 11.8% of the nurses had ever screened for cervical cancer. However, majority (92.2%) were willing to recommend the screening to others.

**Conclusion:**

The respondents of this study had some knowledge of cervical cancer however had low patronage for recommended screening practices. Therefore, strategies must be implemented to increase screening practices among female nurses.

## Introduction

Cervical cancer is an unrestrained growth of the cells of the cervix. The cervix is the lower, narrow part of the uterus, which protrudes into the upper part of the vagina (National Cancer Institute, 2020, American Cancer Society, 2022). All cases of cervical cancer can be attributed to Human Papilloma Virus (HPV) (National Cancer Institute, 2020, WHO, 2022). HPV is the most common viral infection of the reproductive tract. According to WHO, seventy percent (70%) of all cervical cancer cases reported worldwide are caused by two types of HPV, namely, HPV 16 and HPV 18 (WHO, 2022). It has however been estimated that about 56% of cervical cancer cases in West Africa are attributed to HPVs 16 or 18 (Ghana-HPV Information Centre, 2021). Other risk factors of cervical cancer include poor personal hygiene, early marriage and childbearing, high parity, smoking, lack of awareness of the disease, and immunosuppression (Paul et al., 2011, Kashyap et al., 2019).

Globally, cervical cancer is the fourth most common cancer among women. In 2020, over half a million women were diagnosed with cervical cancer, and more than half of these women died from the condition. Unfortunately, most of the diagnosed cases and mortalities of cervical cancer in 2020 occurred in developing countries (Sung et al. 2021). The lack of organized screening and HPV vaccination programs in developing countries contribute to about 90% of cervical cancer cases in these regions (Cohen et al., 2019). In Ghana, it has been estimated that a little over a quarter of a million women are diagnosed with cancer, and more than half of these women die from the condition every year (Ghana-HPV Information Centre, 2021).

Cervical cancer ranks as the second most frequent cancer among women in Ghana between 15 and 44 years of age (Ghana-HPV Information Centre, 2021). Adequate knowledge on cervical cancer is foundational to preventing the condition among women. Healthcare workers, especially nurses in all settings, are responsible for teaching and creating awareness on cervical cancer to women to increase their knowledge on the condition to help reduce the incidence of the condition and the mortality associated with it. Most studies done in other settings have found that health professionals, including nurses, have adequate knowledge about cervical cancer; however, their patronage of screening services which is one of the preventive measures against the disease, is low (Ali et al., 2010, Awodele et al., 2011, Shah et al., 2012, Oche et al., 2013, Gebreegziabher et al., 2016, Dulla et al., 2017, Eze et al., 2018).

Various research studies in Ghana have highlighted the poor knowledge of women about cervical cancer and their low patronage of screening services (Abotchie and Shokar, 2009, Ebu et al., 2014). Limited studies have been conducted in Ghana on nurses' knowledge and screening practices on cervical cancer, even though nurses form the majority of health professionals and are often the first and frequent point of call for patients (Williams et al. 2018). Additionally, statistics on cervical cancer screening in Asikuma Odoben Brakwa, a major district in the Central Region of Ghana, have revealed that only 1.3% of the women screened in 2018 were health workers (Cervical Cancer Screening Unit Annual Report 2018). Thus, this study aimed to assess the knowledge, perception, and screening practices on cervical cancer among female nurses and midwives at a major health facility in the Central Region of Ghana.

## Material and methods

### Study design and setting

The study employed a quantitative, descriptive cross-sectional research design. The study was conducted at Our Lady of Grace Hospital in Breman Asikuma, which is in the Asikuma Odoben-Brakwa District of the Central Region of Ghana. It is a 150-bed health facility and a National Health Insurance Scheme accredited mission hospital located in a farming community in the Central Region. The hospital has an out-patient department, emergency department, medical ward, surgical ward, paediatric ward, maternity ward, operating theatre, public health unit, pathological laboratory, radiology department, pharmacy, physiotherapy department, and a dental department.

### Sample size and sampling technique

The study's sample size was calculated using Yamane's formula. A total of 130 respondents were recruited as the sample size for the study using the convenience sampling technique. However, 102 completed questionnaires were retrieved. The inclusion criteria comprised all registered nurses and midwives at the various wards of the hospital who consented to participate in the study. The study excluded all unregistered nurses and midwives at the hospital, as they work under supervision. In addition, all registered nurses and midwives on annual leave, and those on the wards who did not consent to participate in the study were also excluded.

### Data collection and instrument

The instrument used in data collection was a self-administered questionnaire. The final questionnaire was adapted after pretesting it among ten (10) registered nurses working at St. Francis Hospital in Assin Fosu in the Central region of Ghana. The final questionnaire was in four sections. The first section focused on the socio-demographic information of respondents and was made up of four items. The second and third sections comprised twelve and six items, respectively, and centered on respondents' knowledge of cervical cancer and their perceptions and beliefs on cervical cancer respectively. The fourth section, which contained nine items, obtained information on screening practices and the experiences of respondents.

Data was collected from April 2022 to May 2022 to ensure all participants who worked on different shifts during the week had an equal chance of being selected. Those who participated in the study were given codes to ensure that no respondent answered more than one questionnaire.

### Data analysis

Standard descriptive statistics such as proportions and means were used to summarize the survey data. Data management and analysis were performed with the Statistical Package for Social Science (SPSS), version 20.0.

### Ethical considerations

The study was approved by the Faculty of Health and Medical Sciences of Presbyterian University College, Ghana, and was conducted according to the guidelines of the Declaration of Helsinki. Adequate information was given to the respondents regarding the research objectives, benefits, risks, and voluntary participation in the study. Approval was sought from the study area, and signed informed consent was obtained from the respondents before data collection. The data collected was kept confidential and anonymized. Coding systems were developed to ensure that data sources were identifiable only by the researchers.

## Results

### Socio-demographic data of respondents

The characteristics of the surveyed population are shown in Table 1. The majority of the respondents were under 36 years of age (88.2%), and were Christians (98.0%). More than half of the respondents were married (59.8%) and were registered general nurses (59.8%).

**Table 1:** Distribution of demographic data of respondents

Variables	Frequency (n)	Proportion (%)
**Age**		
20-25 years	19	18.6
26-30 years	37	36.3
31-35 years	34	33.3
36-40 years	11	10.8
46-50 years	1	1.0
Total	102	100.0
**Religious affiliation**		
Christianity	100	98.0
Muslim	2	2.0
Total	102	100.0
**Marital status**		
Single	38	37.3
Married	61	59.8
Divorced	3	2.9
Total	102	100.0
**Professional qualification**		
Community health nurse	3	2.9
Enrolled nurse	7	6.9
Registered general nurse	61	59.8
Registered mental nurse	6	5.9
Registered midwife	25	24.5
Total	102	100.0

### Knowledge of cervical cancer

Information on the knowledge of cervical cancer among nurses surveyed is presented in Table 2. The majority of respondents (99.0%) had heard of cervical cancer. Respondents' sources of information on cervical cancer were the media (57.8%), co-workers (19.6%), friends and family (11.8%), and nursing training school (10.8%).

**Table 2:** Knowledge of cervical cancer

Variables	Frequency	Proportion (%)
**Have you ever heard about Cervical Cancer?**		
Yes	101	99.0
Don't Know	1	1.0
Total	102	100.0
**If yes to question 5, where did you hear about cervical cancer**		
Media	59	57.8
Co-Worker	20	19.6
Learned about it in School	11	10.8
Friends and family	12	11.8
Total	102	100.0
**Who can be affected with cervical cancer?**		
Men	1	1.0
Women	101	99.0
Total	102	100.0
**What age group can be affected with cervical cancer?**		
Children	1	1.0
Adolescent	4	3.9
Adults	84	82.4
All age groups	10	9.8
Don't know	3	2.9
Total	102	100.0
		
**What are the risk factors of cervical cancer?** **Early age of first intercourse**		
Yes	67	65.7
No	35	34.3
Total	102	100.0
**Multiple sexual partners**		
Yes	56	54.9
No	46	45.1
Total	102	100.0
**Increased parity**		
Yes	45	44.1
No	57	55.9
Total	102	100.0
**Immunosuppression**		
Yes	65	63.7
No	37	36.3
Total	102	100.0
**Genital infections**		
Yes	53	52.0
No	49	48.0
Total	102	100.0
		
**What causes Cervical Cancer?**		
Virus	98	96.1
Fungus	1	1.0
Bacteria	3	2.9
Total	102	100.0
**What is the name of the organism?**		
Human Papilloma Virus	93	91.2
Don't know	8	7.8
Squamous cells	1	1.0
Total	102	100.0

When they were asked about who could be affected by cervical cancer, a vast majority of respondents (99.0%) indicated that women are the ones affected by cervical cancer. In addition, about two-thirds of the respondents (65.7%) indicated early age at first sexual intercourse as a risk factor, whereas 54.9% (n=56) said having multiple sexual partners is a risk factor for cervical cancer. More than half (55.9%) of the respondents disagreed that increased parity is a risk factor for cervical cancer. Furthermore, the majority of respondents (63.7%) said immunosuppression is a risk factor for cervical cancer, and 52.0% cited vaginal infections as a risk factor for cervical cancer. In addition, more than half (52.0%) of the respondents agreed that genital infections could be a risk factor for cervical cancer. The majority (96.1%) of respondents said viruses are the causes of cervical cancer, and 2.9% (n=3) said bacteria and fungus (1.0%) are the causes of cervical cancer. Furthermore, the majority (91.2%) of the respondents disclosed that human papillomavirus is the organism that causes cervical cancer, while 7.8% had no idea what the causative organism was, and 1.0% indicated squamous cell carcinoma as the cause of cervical cancer.

Table 3 below outlines respondents' knowledge on the mode of transmission, clinical manifestations, screening centers, and cervical cancer screening methods.

**Table 3:** Knowledge of cervical cancer

Variables	Frequency (n)	Proportion (%)
**How is the causative organism mentioned transmitted?**		
Sexual contact	92	90.2
Non sexual contact	4	3.9
Don't know	6	5.9
Total	102	100.0
		
**What are the presenting symptoms of cervical cancer**		
Abnormal menstrual bleeding	59	57.8
Bleeding between menstrual period	10	9.8
Itching of the vagina	3	2.9
Unpleasant vaginal discharge	5	4.9
Post-menopausal vaginal bleeding	5	4.9
Post coital bleeding or bleeding after sexual intercourse	1	1.0
Don't Know	19	18.6
Total	102	100.0
		
**Do you know any cervical cancer screening centers?**		
Yes	93	91.2
No	9	8.8
Total	102	100.0
		
**Frequently mentioned screening centers**		
Private Hospitals	34	36.6
37 Military Hospital	5	5.4
Trauma Hospital like Winneba	2	2.1
District, Regional and Teaching Hospital	48	51.6
University Hospitals	4	4.3
Total	93	100.0
		
**Which screening methods do you know?**		
Pap smear	60	58.8
Visual inspection with acetic acid (VIA)	32	31.4
Colposcopy	10	9.8
Total	102	100.0

Most respondents (90.2%) agreed that sexual contact was the main mode of virus transmission to the cervix. When asked about the presenting symptoms of cervical cancer, 57.8% of the respondents stated abnormal menstrual bleeding, 18.6% indicated they had no idea, 9.8% revealed bleeding between menstrual period, 4.9% said unpleasant vaginal discharge, 4.9% cited post-menopausal vaginal bleeding, 2.9% indicated itching of the vagina and only 1.0% revealed post-coital bleeding or bleeding after sexual intercourse.

Furthermore, the majority of the respondents (91.2%) stated that they were aware of cervical cancer screening centers, with 51.6% of them indicating that all district, regional, and teaching hospitals are screening centers, while the rest mentioned private hospitals (36.6%), 37 Military Hospital (5.4%), Trauma Hospitals (2.1%) and University Hospitals (4.3%). In addition, most of the respondents (58.8%) revealed that a Pap smear is a screening method they are familiar with, followed by visual inspection with acetic acid (31.4%) and colposcopy (9.8%).

### Perceptions and beliefs on cervical cancer

Table 4 shows respondents' perceptions and beliefs on cervical cancer. Most respondents (67.6%) believed that cervical cancer is incurable. Also, the majority of respondents (97.1%) perceived cervical cancer to be preventable and they believed it could be prevented through vaccination (52.9%), education (44.1%), and screening (2.9%). Furthermore, only 39.2% of the respondents perceived themselves as at risk of acquiring cervical cancer. However, a few of the respondents (18.9%) could not tell whether they were at risk or not.

**Table 4:** Perceptions and beliefs on cervical cancer

Variables	Frequency	Proportion (%)
**Can cervical cancer be cured?**		
Yes	29	28.4
No	69	67.6
Don't Know	4	3.9
Total	102	100.0
**Is cervical cancer preventable?**		
Yes	99	97.1
No	1	1.0
Don't Know	2	2.0
Total	102	100.0
**Frequently mentioned cervical cancer preventive measures**		
Education	45	44.1
Vaccination	54	52.9
Screening	3	2.9
Total	102	100.0
**Do you think you are at risk of getting cervical cancer?**		
Yes	40	39.2
No	43	42.2
Don't know	19	18.6
Total	102	100.0
**Do you think screening is effective in reducing the incidence of cervical cancer?**		
Yes	98	96.1
No	3	2.9
Don't know	1	1.0
Total	102	100.0
**Do you think poor personal hygiene causes cervical cancer?**		
Yes	23	22.5
No	50	49.0
Don't know	29	28.4
Total	102	100.0

Moreover, most of the respondents (96.1%) believed that screening for cervical cancer effectively reduces the incidence of the disease. In addition, 49.0% of the respondents did not perceive poor personal cleanliness as one of the causes of cervical cancer.

### Screening practices and experiences

Table 5 below depicts cervical cancer screening practices and the experiences of the nurses. The majority of the respondents (88.2%) had never been screened for cervical cancer due to respondents' lack of self-confidence (51.1%), high cost of screening (36.7%), belief that they were not at risk (10.0%), not knowing about screening (1.1%), and the lack of screening centers (1.1%). More than half of the respondents (51.0%) agreed that cervical cancer screening should be done yearly. However, 29.4% (n=30) of the respondents did not know how often a woman should be screened for cervical cancer. Most (56.9%) respondents opined that they would voluntarily screen for cervical cancer if it was free.

**Table 5:** Screening practices and experiences

Variables	Frequency	Proportion (%)
**Have you ever screened for cervical cancer?**		
Yes	12	11.8
No	90	88.2
Total	102	100.0
**If yes to question 23 above, how many times have you screened?**		
Once	9	75.0
Twice	3	25.0
Total	12	100.0
**If no to question 23 above, why have you not screened for cervical cancer?**		
Don't know about screening	1	1.1
High cost of screening	33	36.7
Unavailability of screening center	1	1.1
I feel shy due to the procedure	46	51.1
I don't think i am at risk	9	10.0
Total	90	100.0
**How often should one be screened?**		
Once a year	52	51.0
3 years	14	13.7
5 years	5	4.9
When symptoms appear	1	1.0
Don't know	30	29.4
Total	102	100.0
**Will you voluntarily screen if it is free?**		
Yes	58	56.9
No	11	10.8
Don't know	33	32.4
Total	102	100.0
**Will you accept screening by a male provider?**		
Yes	26	25.5
No	76	74.5
Total	102	100.0
**Will you recommend screening to a client or friend?**		
Yes	94	92.2
No	6	5.9
Don't know	2	2.0
Total	102	100.0
**How much are you willing to pay for screening?**		
10-20 cedis	80	78.4
30-50 cedis	20	19.6
60-100cedis	2	2.0
Total	102	100.0
**If you have screened before, how was the experience?**		
It was interesting and good at same time.	1	8.3
Not bad	1	8.3
Uncomfortable	4	33.3
Painful	6	50.0
Total	12	100.0

The majority of respondents (74.5%) said they would not accept cervical cancer screening from a male clinician. Additionally, most respondents (92.2%) would recommend screening to a client or friend. Respondents were willing to pay between 10 and 20 Ghana Cedis (1.2 – 2.4 US Dollars) (78.4%), 30 to 50 Ghana Cedis (3.6 – 5.9 US Dollars) (19.6%), and 60 to 100 cedis (7.1 – 11.8 US Dollars) (2.0%) for screening. Out of the 12 respondents who had been screened for cervical cancer, 50.0% (n=6) revealed that screening was painful, 33.3% (n=4) indicated they were uncomfortable, one respondent said it was interesting, and she felt good, and the other respondent stated the experience was not bad.

## Discussion

The present study revealed that nurses have a fair knowledge of cervical cancer, and this finding is consistent with the many studies conducted in other settings where health professionals including nurses have adequate knowledge of cervical cancer (Dulla et al., 2017, Eze et al., 2018, Gebreegziabher et al., 2016, Shah et al., 2012, Ali et al., 2010, Awodele et al., 2011). In the current study, the nurses' fair knowledge of cervical cancer, could be as a result of their sources of information of which majority said was the media. Generally, nurses' main source of information on cervical cancer should be during their course of training in school as they will acquire adequate information on the condition and not depend on other sources. Thus, more nursing training schools should include cervical cancer in their curriculum and workshops should be organized for healthcare workers on cervical cancer at the various health facilities (Urasa and Darj, 2011).

The current study demonstrated that respondents knew cervical cancer risk factors. The respondents cited early age at first sexual intercourse, multiple sexual partners, immunosuppression, and genital infections as risk factors for cervical cancer. This finding is similar to findings of other studies in other settings where respondents identified early age at first sexual intercourse, and multiple sexual partners as cervical cancer risk factors (Ertem, 2009, Paul et al., 2011). In addition, nurses in this current study identified Human Papilloma Virus as the organism that causes cervical cancer and recognized that the mode of transmission of the virus is via sexual intercourse. These findings are consistent with other studies which identified HPV infection as the most important risk factor for cervical cancer and also recognized sexual contact as the major mode of transmission of the virus (Paul et al., 2011, Urasa and Darj, 2011). Furthermore, nurses in this current study knew the signs and symptoms of cervical cancer. They cited abnormal bleeding, unpleasant vaginal discharge, post-menopausal bleeding, and inter-menstrual bleeding as the signs and symptoms of cervical cancer. This finding is consistent with similar studies in other settings where respondents identified abnormal bleeding, post-menopausal bleeding among others as signs and symptoms of cervical cancer (Urasa and Darj, 2011, Ertem, 2009).

This present study demonstrated that nurses knew about various screening centers and cervical cancer screening methods such as Pap smear, Visual Inspection with Acetic acid (VIA) and colposcopy. These findings contrast with the findings of a study conducted in Ghana by Ebu et al. (2014) where most respondents did not know the screening methods of cervical cancer and where to assess them. This suggests that nurses know about this information because they are health workers.

Although nurses in this current study knew about HPV, its mode of transmission, risk factors, screening methods and centers, and the signs and symptoms of cervical cancer, only a few of them perceived themselves as at risk of acquiring cervical cancer. Hence, the majority had never been screened for cervical cancer, which is consistent with many studies conducted among healthcare workers on their screening practices (Ertem, 2009, Awodele et al., 2011, Dulla et al., 2017). Nurses in the current study do not go for cervical cancer screening because they are shy of the procedure as the procedure perhaps, is being provided by male health professionals. Others respondents do not patronize screening services because of high cost of Pap smear. Few other respondents perceived themselves as not at risk of cervical cancer because, perhaps they are not sexually active hence, do not go for screening services. Very few of the respondents said they do not know where to access the screening services. In similar studies conducted by Ebu et al. (2014) and Dulla et al. (2017) some of the respondent's reasons for not screening included lack of information on screening methods, fear of the result, shyness due to the procedure, inability to afford the cost of screening and religious beliefs. The reasons why nurses in the present study do not screen for cervical cancer seem to suggest that they do not serve as good role models for patients and/or other women in cervical cancer screening. The above findings also support the assertion that adequate knowledge of cervical cancer may not necessarily result in screening for the condition.

Furthermore, most nurses in this present study demonstrated their willingness to screen for the condition if it were at no or low cost. This suggests the need to incorporate cervical cancer screening (Pap smear) into the services covered by Ghana's national health insurance scheme. In addition, the majority of nurses in this study stated that they would not accept screening from a male health provider due to shyness. This implies that more female health workers should be trained in carrying out screening procedures. Again, the few nurses in this present study who were screened expressed their experiences as being painful, uncomfortable and no negative experience with the procedure. This is consistent with the study by Ebu et al. (2017), which found that most respondents who screened for cervical cancer mentioned that a Pap smear was painful and embarrassing. However, the majority of the nurses in this study were willing to recommend cervical screening to a friend or a client.

Most nurses in this present study believed that cervical cancer is incurable. However, the majority of them perceived that it could be prevented via vaccination, education and screening. This finding is similar to the finding of a study carried out by Dulla et al. (2017) among nurses in Southern Ethiopia, which identified that cervical cancer could be prevented. Most nurses in this current study did not consider cervical screening as a preventive measure. It is apparent that this perception resulted in the low cervical cancer screening practices among them. That notwithstanding, the majority of them believed that cervical screening is effective in reducing the incidence of cervical cancer.

In conclusion, this study provides further evidence that even though nurses know about cervical cancer, and its screening procedures and centers, most of them do not get screened. Their reasons for low patronage of screening services include shyness, high cost of Pap smear, and belief that they are not at risk.

Despite the utility of this study's findings, one must consider some limitations of the study. This study being a cross sectional study, the findings only give a snapshot of the situation at the time of the study. It is therefore, possible that the situation may have changed since the study. Thus, a repeated study may help describe the current trend of nurses and midwives' screening practices for cervical cancer. This will undoubtedly improve the health and wellbeing of not only female nurses but all women in Ghana at large.
